# Effect of Soil Environment on Species Diversity of Desert Plant Communities

**DOI:** 10.3390/plants12193465

**Published:** 2023-10-02

**Authors:** Jie Sun, Nai’ang Wang, Zhenmin Niu

**Affiliations:** 1 College of Ecology and Environment, Xinjiang University, Urumqi 830017, China; sunjxju@126.com; 2 College of Earth and Environmental Sciences, Center for Glacier and Desert Research, Lanzhou University, Lanzhou 730000, China; niuzhm@nxu.edu.cn; 3 School of Geography and Planning, Ningxia University, Yinchuan 750021, China

**Keywords:** species diversity, Alxa desert, desert plants, soil properties, environmental response

## Abstract

Desert ecosystems possess an astonishing biodiversity and are rich in endangered species. This study investigated characteristics of species diversity and soil environmental factors in three major deserts of China’s Alxa Plateau. The Alxa Desert included 183 plant species belonging to 109 genera and 35 families. The highest numbers of plant species belonged to the Compositae, Gramineae, and Chenopodiaceae families. The research area belongs to the semi-shrub and small semi-shrub deserts in temperate deserts. Species diversity was low, with the Shannon–Wiener index (*H*′) of shrub-herb = shrub > herb > tree. The Pielou evenness index (*E*) of shrub herb vegetation was the lowest, indicating more enriched species and fewer sparse species in the community, and that these types of vegetation had the characteristics of rich and obviously dominant species. Redundancy analysis (RDA) and correlations between the comprehensive plant community biodiversity index and soil factors indicated that soil-available phosphorus (NP), organic matter (SOM), and electrical conductivity (EC) had significant impacts on community species diversity. The herbaceous shrub community exhibited the highest *H*′, Simpson index (*D*), species richness index (*S*), soil moisture (SW), and soil nutrients. Planting *Calligonum mongolicum*, *Ephedra membranacea*, *Artemisia annua*, and *Phragmites australis* to form a typical desert shrub community for community diversity protection is recommended to effectively protect and restore desert ecosystems.

## 1. Introduction

The study of plant species diversity patterns and their influencing factors form the basis of ecology and conservation biology [1,2]. Species diversity is the basis for plants to maintain the structure, function, and stability of ecosystems [3]. Changes in biodiversity affect ecosystem services by altering the function and stability of ecosystems [4], and maintaining ecosystem services requires a high degree of plant diversity [5]. Various hypotheses, including the energy hypothesis, have been proposed to explain the patterns of distribution of species diversity. Different forms of energy have different impacts on the mechanisms of species diversity. The hypothesis suggests that energy changes controlled the species diversity [6]. In addition, changes in precipitation can alter the functionality of ecosystems, thereby altering the pattern of plant species richness [7,8]. In recent years, researchers have attempted to explore the relationship between plant species diversity and the soil environment in ecosystems [9,10]. However, the results of these studies have not been consistent, especially in plant communities in natural habitats [11] and arid areas [12].

Approximately 3-billion people worldwide reside in desert ecosystems (IPCC, 2019) [13], which account for approximately 22% of the global land area [14]. Desert ecosystems in northern China are home to various endangered plants [15]. Due to the potential significant impact of desertification on China’s ecology and food security, the Chinese government has launched multiple desertification prevention and control measures to improve vegetation conditions [16,17,18]. To support these measures in desert ecosystems, the study of species diversity is extremely important, and funds for protection and research on desert ecosystems are lacking. Therefore, it is urgent for humans to face up to the study of plant species diversity in the desert [19]. Desert ecosystems are more barren than other ecosystems, and plant growth is limited by environmental factors such as soil nutrients [20]. The limited and uneven distribution of water and nutrients leads to plants appearing in strips or patches [21]. The fertilizer islands are formed under shrub vegetation, thereby promoting the spatial variability of soil factors [22,23,24]. In the desert, do tree and herb fertilizer islands have an effect? Conversely, how do soil environmental factors affect plants? There is no consensus for the time being. Studying the interaction between plants and soil factors is helpful for the restoration and stability of vegetation in desert ecosystems. In the context of global change and the rapid reduction of species diversity, we need to explore the species diversity mechanism to protect it in deserts. At present, many studies have focused on biodiversity-rich hotspots, while remote and species-poor areas such as deserts lag behind hotspots [2,25]. Understanding the impact of desert environments on biodiversity is a prerequisite for diversity conservation [26]. Which soil environmental factors have the greatest impact on desert biodiversity? How do they affect biodiversity patterns?

There are three major deserts in the Alxa Plateau: Badain Jaran, Tengger, and Ulan Buh Deserts, which belong to the northern sand area of China with extremely important ecological protection and are representative areas for better desert ecological protection, breeding typical arid desert plants and plenty of endemic species [27]. Ecosystems are highly fragile to the environment [28] due to harsh environments (gale disasters, severe soil salinization, poor availability of soil nutrient and moisture), and it will be important to understand the biodiversity response to climate change. Species diversity research is the core issue of desert ecosystem ecology research. It focuses mainly on three aspects: the protection of desert ecosystem, reconstruction, and sustainable development. The three deserts have a large geographical area, and environmental factors such as soil are heterogeneous. This provides a suitable location for studying the spatial relationships between soil properties and species diversity.

We considered the Alxa Desert, China as the research object and studied the species diversity and its influencing factors. The main objectives of the study were: (1) To determine the distribution of species diversity of the three desert plant communities in the main body of the Alxa Plateau. (2) To analyse the correlations between diversity indices of typical desert communities and soil environmental factors to provide a decision-making basis for the effective protection and restoration of desert ecosystems.

## 2. Results

### 2.1. Community Species Diversity and Species Composition

The plant community composition in the study area was relatively simple, with a total of 183 plant species belonging to 35 families and 109 genera (Table 1), among which 22 rare species appeared only twice or less in all quadrats, as shown in Table 1. Among them, Compositae, Poaceae, and Chenopodiaceae were the most abundant, accounting for 50.29% of the total species, followed by Leguminosae. The sample survey of the Badain Jaran Desert plant species identified 26 families, 73 genera, and 109 species; the Tengger Desert sample survey produced 27 families, 83 genera, and 118 species; and in Ulan Buh Desert, 12 families, 39 genera, and 52 species were identified. See Supplementary Materials File (Table S1) for detailed species list.

### 2.2. Species Diversity Index and Distribution

A total of 174 representative plots and 479 quadrats were set to study the diversity index, evenness, and richness. Of these, 101 samples were selected from the Badain Jaran Desert, 56 from the Tengger Desert, and 17 from the Ulan Buh Desert. Indices of characteristics of diversity, including the Shannon–Wiener index (*H*′), Simpson index (*D*), Pielou evenness index (*E*), importance value (*IV*), Species richness index (*S*) of the herb, shrub, and other vegetation layers were calculated for the three deserts.

The plant community species richness index had a minimum value of 1 and a maximum of 31 for the Alxa Desert. The species richness of the plant community in the Badain Jaran Desert was low, with an average value of 4.41. The species richness of a single quadrat was mostly less than five. The species richness of the plant community was slightly higher for the Tengger and Ulan Buh Deserts than that of the Badain Jaran Desert; although the richness index reached 30, the overall richness index was still low.

The highest value observed for the Shannon–Wiener index of species diversity among the Alxa Desert plant communities (2.62) appeared in the *Phragmites* association of the Badain Jaran Desert. Values of the index greater than 2 were more commonly observed in the Tengger Desert, and the observed lowest value was observed mostly in the Badain Jaran Desert. The maximum value observed for Simpson’s index among the Alxa Desert plant communities (0.92) was observed in the Badain Jaran Desert. The maximum evenness index value observed was one, which was observed in the Badain Jaran Desert, and the minimum observed value was 0.01, which was observed in the *Astragalus mongholicus*—Mongolica fruit community in Tengger Desert.

The Shannon–Wiener, Simpson index, and species richness of plant species in the Tengger Desert were relatively high, and the evenness was relatively low. The Shannon–Wiener index, Simpson index, and species richness of the Badain Jaran Desert were lower than those of the 174 plots in the entire Alxa Desert, and the evenness was higher than those of other major deserts and higher than the overall value observed for the Alxa Desert (Table 2).

Considering the species diversity of different life forms (Table 3), the Shannon–Wiener index of shrubs and shrub–herbs was 0.79, the Simpson index was 0.39, the richness index was 5.40 and 5.97, respectively, which were the highest), and the Shannon–Wiener and Simpson indices of trees were the smallest. The evenness indices of shrubs and shrubs–herbs were 0.56 and 0.48, respectively. The evenness index of shrubs–herbs < shrubs < herbs and trees.

### 2.3. Relationship between Species Diversity and Soil Abiotic Factors

**Figure 1:**
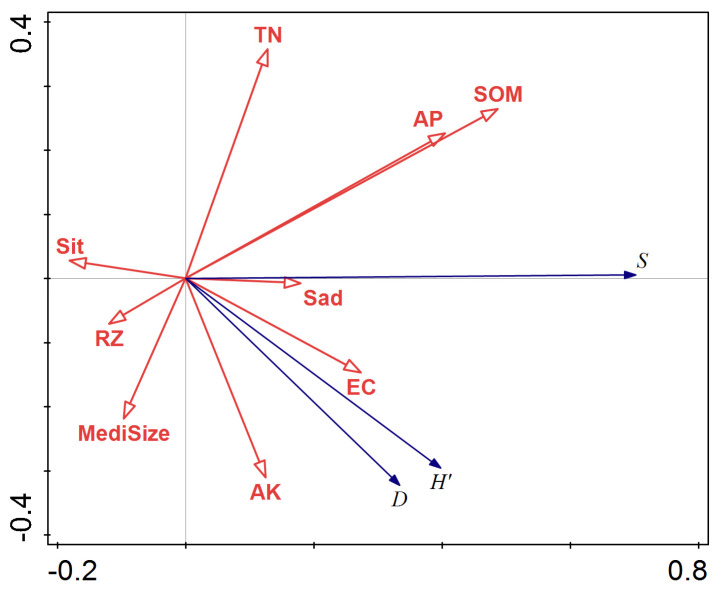
Redundancy analysis (RDA) ordination of species diversity index and soil factors for axis1 and axis2. (*S* = Species richness indexes; *H*′ = Shannon–Wiener index; *D* = Simpson index; Sit = powder content; Sad = sand content; medi size = median particle size; TN = total nitrogen; SOM = soil organic matter; EC = electrical conductivity; AK = available potassium content; AP = available phosphorus; RZ = soil bulk density).

Fifty-eight representative plots were set to analyze their relationship with the soil environment. The data of soil environment were determined experimentally (Table 4). The RDA ordination results are shown in Figure 1. The redundancy analysis (RDA) of species diversity index and soil factors indicated that the first and second axes, particularly the first axis, could explain most of the diversity changes. Soil organic matter (SOM), total nitrogen (TN), and available phosphorus (AP) in the soil profile were positively correlated with the first axis, while the correlation between SOM and diversity index was significant. The powder content of soil (Sit), soil bulk density (RZ), and the median particle size of soil (Median size) was negatively correlated with the first axis, with correlation coefficients of −0.18, −0.25, and −0.19, respectively. In summary, the factors that had the greatest impact on species diversity were SOM, TN, and AP, suggesting that observed species diversity was a consequence of the combined effects of these three factors.

**Figure 2:**
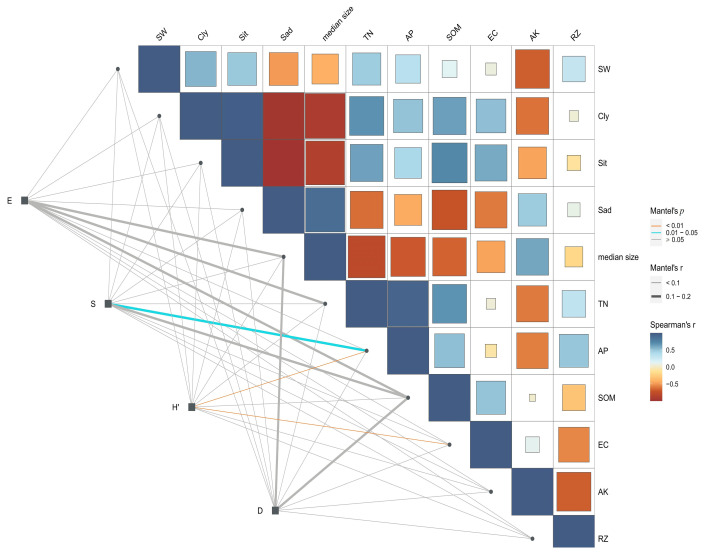
Spearman correlation analysis of plant communities and soil factors in Alxa Desert (*E* = Pielou evenness index; *S* = Species richness indexes; *H*′ = Shannon–Wiener index; *D* = Simpson index; SW = soil moisture content; Cly = clay content; Sit = powder content; Sad = sand content; Median size = median particle size; TN = total nitrogen; NP = available phosphorus; SOM = soil organic matter; EC = electrical conductivity; AK = available potassium content; AP = available phosphorus; RZ = soil bulk density).

Shannon–Wiener diversity and soil-available phosphorus content showed a relatively strong positive correlation, followed in magnitude by the correlations between Shannon–Wiener diversity and conductivity. The Simpson index showed positive correlations with median size and electrical conductivity (EC). The Simpson index was also positively correlated with soil-available potassium content (AK), soil moisture content (SW), and AP (Figure 2). These results suggest that the species diversity of plant communities in desert ecosystems were primarily affected by the available phosphorus content and electrical conductivity in the soil.

## 3. Discussion

### 3.1. Species Composition

According to the Community Survey, five vegetation types (desert, grassland, meadow, shrub, and deciduous broad-leaved forest) were distributed in the Alxa Desert. Among the plants of temperate desert vegetation in China, hyperxeric semi-shrub and shrub species were the most common [29,30]. They adapt to various harsh conditions in the desert and form a variety of desert plant communities. Desert vegetation was also the main type in the Alxa Desert, and its vegetation formation primarily belonged to the semi-shrub, small semi-shrub desert, and shrub desert in temperate deserts groups. Typical desert plant species such as those of the Compositae, Gramineae, and Chenopodiaceae families were the most abundant, forming the desert communities of *Artemisia desertorum* + *Calligonum mongolicum*, *Ephedra przewalskii*, and *Artemisia desertorum* + *Artemisia ordosica*.

Among the three deserts in the Alxa Plateau, the Tengger Desert had the most abundant species of higher plants, covering the largest number of families, genera, and species. This pattern is attributable to the construction of artificial sand-fixing vegetation in the Shapotou National Nature Reserve. Restoration of species diversity has transformed the original relatively simply structured sand-fixing vegetation into a desert ecosystem with a relatively complex structure, composition, and function [31]. The numbers of plant species, families, and genera in the Badain Jaran Desert were second to those of the Tengger Desert, and those in the Ulan Buh Desert were relatively few.

### 3.2. Species Diversity

The Simpson and Shannon–Wiener indices are commonly used in community species diversity research and can characterise community evenness and richness, while the Shannon–Wiener index represents heterogeneity, is positively correlated with diversity, and is used to characterise species diversity. The more uniform the species distribution in plant community, the greater the Shannon–Wiener index and the higher the species diversity. The Simpson index was negatively correlated with other diversity indices, which characterized the role and status of dominant species. The Pielou evenness index reflects the uniformity of the abundance, coverage, biomass, and other indicators of different species in the community.

Theoretically, the richer the species diversity in a community, the greater the Shannon–Wiener index, and the Shannon–Wiener index distribution of different vegetation life-form species in the Alxa Desert conformed to this law. The Shannon–Wiener index of each vegetation layer was ordered as follows: shrub–herb = shrub > herb > tree (Table 3). The evenness index indicates the uniformity of the distributions of abundance of different species in a community. Among the vegetation life-forms of the study area, the evenness index of shrub–herb vegetation was the lowest, which indicated that there were more enriched species and fewer sparse species in the community. That is, this type of vegetation had the characteristics of rich species and evidently dominant species. In general, the level of species diversity index in the study area was low; the diversity index of shrubs and shrubs–herbs was the highest, and the evenness index was low, indicating the characteristics of plant communities in desert ecosystems.

The Shannon–Wiener index, Simpson index, and species richness of plant species in the Tengger Desert were relatively higher than those among the other two major deserts of Alxa, and the evenness was relatively low. The Shannon–Wiener index, Simpson index, and species richness of the Badain Jaran Desert were low, and the evenness was high. This is also consistent with the observations (given in Section 2.1) that the plant species in the Tengger Desert were the most abundant, followed by those in the Badain Jaran Desert, and the abundance, coverage, biomass, and other indicators of different species in the Badain Jaran Desert community were relatively uniform, while the dominant species of the Tengger Desert plant community were evident.

### 3.3. Impact of Soil Factors on Community Species Diversity

The soil moisture content of the shrub–herb community was the highest (4.1%), and the soil nutrient status of the shrub–herb community was second only to that of the herbaceous community, which included some *Phragmites australis* distributed around the lakes and dry lake basins.

Consistent with the research results of Li et al. [2], there was a certain correlation between community diversity and soil factors. The RDA rankings and correlations between the comprehensive plant community biodiversity index and soil factors indicated that soil-available phosphorus, organic matter, and electrical conductivity were the soil factors that significantly impacted on community species diversity.

Similar to the results of Zhijun et al. [32], the content of soil phosphorus, especially soluble and easily desorbed available phosphorus, was considered to be an important limiting factor in arid desert ecosystems. As it played an indispensable role in the biochemical reaction and nutrient cycle of plants, this study also found that available phosphorus also has a greater impact on the distribution of species diversity. Water and salinity are the main limiting environmental factors in desert ecosystems [33]. Plant growth, nutrient cycling, and biological functions are significantly affected by soil water content (SW) and salinity [34]. However, due to the significant correlation between SW and salinity [35], it has been suggested that salinity should be added to the minimum soil attribute dataset to predict the spatial variability of desert species diversity, and soil water content should be removed [36]. This is consistent with the fact that EC has a great influence on the distribution of plant species diversity in this paper. Organic carbon is the support of soil fertility [37,38]. Our study confirmed the important impact of SOM on plant diversity. Different from previous studies [39,40], the effect of soil nitrogen content on plant species diversity is not reflected. However, the effect of available potassium is quite different in different study areas [41,42], which may be related to the unique landform and plant species in the study area. In addition, climate change and human activities have a crucial impact on the distribution of species diversity [43,44,45]. In this study, the impact of sand control engineering on communities with stronger stability due to climate and human planting was not considered, which needs further consideration.

Considering soil water, nutrients, and species diversity, the shrub–herbaceous community showed the highest Shannon–Wiener index, Simpson index, richness index, and soil environmental conditions. Due to its high community species diversity and favorable soil environment, considering the composition of local plant communities, planting *Calligonum mongolicum*, *Ephedra przewalskii*, *Artemisia desertorum*, and *Phragmites australis* to form *Artemisia desertorum* + *Calligonum mongolicum*, *Artemisia desertorum* + *Ephedra przewalskii*, *Artemisia desertorum* + *Artemisia ordosica* desert, and *Phragmites australis* meadows can effectively protect and restore desert ecosystems.

Understanding the impact of environmental factors on biodiversity is a prerequisite for biodiversity conservation. Then, managers can start to improve soil environmental conditions by increasing the content of these soil factors in an attempt to increase the biodiversity distribution of desert nature reserves and even the entire desert and conduct ecological restoration and reconstruction.

## 4. Materials and Methods

### 4.1. Study Area

Alxa Plateau is located in the Inner Mongolia Autonomous Region, west of Helan Mountain and Wolf Mountain, east of Ma Manshan Mountain, north of Hexi Corridor, and south of the border between China and Mongolia, covering an area of 300,000 km^2^. The terrain is high in the south and low in the north, with an altitude of about 1000–1500 m, and the ground does not fluctuate much. On the surface, in the east of the plateau, the desert is widespread, and in the west, the Gobi predominates. The main parts of the plateau are the Badain Jilin, Ulan Buh, and Tengri deserts, all of which are dominated by mobile dunes [46]. This is one of the most severe desertification areas in China [47] and one of the most important desert areas in China, and perhaps the world. Within the region, the northeast–southwest-trending Yabulai Mountains (altitude 1800–2000 m) are flanked by the Tengger and Badain Jaran Deserts. In terms of geographical distribution, the Alxa Plateau is located on the Alxa Desert in the desert subregion of Central Asia. The annual precipitation of the Alxa Plateau is 50–150 mm and increases from the northwest to the southeast. Water resources are scarce, and surface water resources are mainly inland rivers, including the Hei and Yellow Rivers [48]. The three deserts are rich in groundwater. The Active Accumulated Temperature is about 3000 °C. The region is located within the desert flora region of Asia and has sparse vegetation with four main subtypes: sandy vegetation, typical desert vegetation, grassland deserts, and desert grassland [28]. This study investigated the Alxa Desert, composed of three major deserts (namely, the Badain Jarin, Tengger, and Ulan Buh Deserts) distributed in the Alxa Plateau. The study area spanned 37°27′ N–42°12′ N, 99°23′ E–107°20′ E.

### 4.2. Plant Community Survey

**Figure 3:**
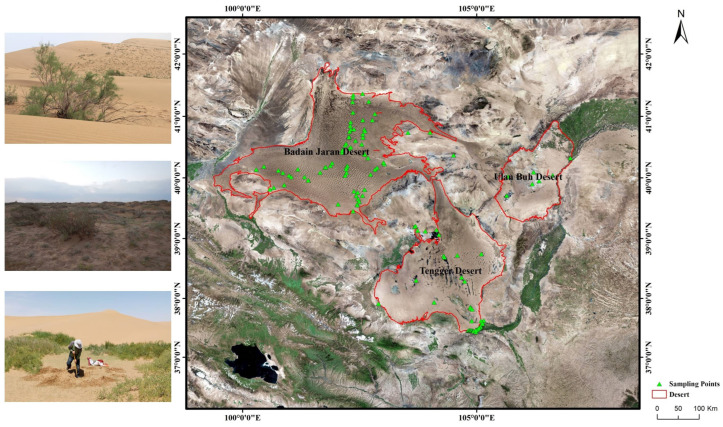
Study area: Three deserts of the Alxa Plateau, China.

Vegetation surveys were conducted in the Badain Jaran, Tengger, and Ulanbuhe Deserts from 2009 to 2016 using the quadrat survey method. Plots were set up for different plant communities according to the experimental method of ecology of plant populations [49,50], and 2–3 replicate quadrats were randomly chosen at each plot, which contained as many plant species as possible. The area of the herbaceous quadrats was set to be 1 m × 1 m, and the range of the shrub quadrats was 10 m × 10 m. The distribution of the survey plots is shown in Figure 3. In the three deserts of Alxa, 174 plots and 479 quadrats were surveyed to investigate the distribution of plant species and the layout of species diversity, collect environmental records within the sampling range. Known the plant distribution status, 58 representative plots were set, and soil samples with different characteristics for typical plant communities were collected to analyze the relationship between plant diversity and soil environment.

For plant communities, the community cover, plant species, community name, and quadrant area were recorded, as were the type, number, tree height, and breast diameter of all trees in the quadrant, and the type, number, cover, height, and crown breadth of the shrub species. Missing species outside the sample were sought and their names recorded. In terms of quality control, during the sample data collation, complete and reasonable sample data were available and included in the statistical scope. Environmental characteristics, including latitude and longitude, altitude, slope, landform, distance from lake/spring, and habitat, were recorded. At the same time, stratified profile sampling was performed near the quadrat for the measurement of the grain size, water content, bulk density, specific gravity, organic matter, available P, available K, total N content, and total salt content of the soil samples.

### 4.3. Investigation and Measurement of Soil Properties

A total of 2313 layered soil samples were collected from the surface (0–20 cm), and 20–50 cm from the plant sample squares using plum blossom sampling. Fresh soil was weighed and brought back to the laboratory to dry to test SW. The remaining soil samples were pretreated by air drying, screening, and subpackaging. Layered soil RZ, particle size, SOM, TN, NP, AK, and EC were determined experimentally. SW was tested by the dry weight method; RZ was obtained by ring knife sampling, drying, and weighing; TN in the soil was obtained by KDN–103F distillation nitrogen fixation device experiments; NP in the soil was measured by a 723 type spectrophotometer; AK in soil samples was measured by the flame photometer method; SOM was measured by potassium dichromate method; and the sand particle size was tested by a Mastersizer 2000 laser particle size analyser. EC was tested using a conductivity meter and a Bresle patch.

### 4.4. Biodiversity Index

The data in quadrants were converted into community species importance values and species diversity indices, including species uniformity, richness, Shannon–Wiener index, and Simpson index, to obtain the distribution law of desert plant diversity for the Alxa Desert. All diversity calculations were performed using R version 3.5.2.

#### 4.4.1. Importance Value of Species (*IV*)

*IV* refers to the relative ecological importance of a species [15]. Many calculation methods for *IV* exist. Usually, indicators are selected from the relative height, coverage, frequency, abundance, and others of the sample plot for calculation in general field investigations. Because the vegetation in the study area is often buried by aeolian sand, relative cover, relative height, and relative frequency were used to calculate the Importance Values of species [51]. The formula used is as follows:

*IV* = (*Hr* + *Cr* + *Fr*)/3 (1)

where *Hr*, *Cr*, and *Fr* are respectively relative height, coverage, and frequency.

#### 4.4.2. α Species Diversity Index

α species diversity is species diversity within a community. It reflects the relative abundance of species, uniformity index, number of species, biodiversity index, and the distribution characteristics of species number and composition. To characterize α species diversity, the Shannon–Wiener index [52], Simpson index [53], species richness, and evenness index [54] were calculated [55]. The formulae are as follows:

(2) $$\mathrm{Shannon–Wiener index:}\;H^{\prime }=-\sum_{i=1}^{n}P_{i}\mathrm{In}P_{i}$$

(3) $$\mathrm{Simpson}\;\mathrm{index}:\;D=1-\sum_{i=1}^{n}P_{i}^{2}$$

Species richness indexes: *S* = *n* (4)

Pielou evenness index: *E* = *H*′/ln*S* (5)

where *n* is the total number of species in the survey plot, *P_i_* is the proportion of the abundance of the *i*th species in the total abundance, and *S* is the total number of species in the community.

#### 4.4.3. Statistical Analysis

An analysis matrix comprising the species diversity index and soil factors was established. The response relationship between the community species diversity index and soil environmental factors was analysed using t-tests and bi-ordered graphs made in Canoco 5.0, which produced an RDA ordination analysis result map composed of plant species diversity arrows, environmental factor arrows, and circle symbols, showing the correlations between community distribution and community species diversity and environmental variables [56].

Spearman correlation analysis of the Shannon–Wiener, Simpson index, evenness, species richness, and soil factors of the Alxa Desert plant community was conducted in the selected sample area. Spearman correlation and result graph were produced using R version 3.5.2.

## 5. Conclusions

In summary, this article studies the relationship between plant community species diversity and soil factors in the Alashan Desert ecosystem. It found that the highest numbers of plant species belonged to the Compositae, Gramineae, and Chenopodiaceae families. They are mainly xerophytic- and saline–alkali-tolerant plants. Soil-available phosphorus, organic matter, and electrical conductivity were the soil factors that significantly impacted community species diversity. The results of this study suggest the planting of *Calligonum mongolicum*, *Ephedra przewalskii*, *Artemisia desertorum*, and *Phragmites australis* to form *Artemisia desertorum* + *Calligonum mongolicum*, *Artemisia desertorum* + *Ephedra przewalskii*, *Artemisia desertorum* + *Artemisia ordosica* desert, and *Phragmites australis* meadows is needed to protect and restore desert ecosystems.

## Figures and Tables

**Table 1:** Plant species distribution in Alxa deserts.

Families	Genera	Species	Percentage of Total Species (%)
Compositae	21	40	21.86
Chenopodiaceae	13	29	15.85
Poaceae	17	24	13.11
Leguminosae	8	19	10.38
Zygophyllaceae	5	10	5.46
Polygonaceae	4	5	2.73
Liliaceae	2	4	2.19
Apocynaceae	3	4	2.19
Salicaceae	2	4	2.19
Asclepiadaceae	2	4	2.19
Ephedraceae	1	3	1.64
Cyperaceae	2	3	1.64
Boraginaceae	2	3	1.64
Plumbaginaceae	2	2	1.09
Tamaricaceae	2	2	1.09
Verbenaceae	2	2	1.09
Rosaceae	2	2	1.09
Solanaceae	1	2	1.09
Caryophyllaceae	2	2	1.09
Convolvulaceae	1	2	1.09
Iridaceae	1	2	1.09
Primulaceae	1	1	0.55
Labiatae	1	1	0.55
Elaeagnaceae	1	1	0.55
Geraniaceae	1	1	0.55
Ranunculaceae	1	1	0.55
Casuarinaceae	1	1	0.55
Vitaceae	1	1	0.55
Umbelliferae	1	1	0.55
Cruciferae	1	1	0.55
Juncaginaceae	1	1	0.55
Asparagaceae	1	1	0.55
Papaveraceae	1	1	0.55
Ulmaceae	1	1	0.55
Bignoniaceae	1	1	0.55
All	109	183	100

**Table 2:** Characteristics of plant communities in three deserts of the Alxa Plateau, China. The data are the average value of different diversity index (mean ± SD).

	Number of Plots	*H*′	*D*	*S*	*E*
Badain Jaran Desert	101	0.71 ± 0.52	0.36 ± 0.24	4.41 ± 3.84	0.5 ± 0.29
Tengger Desert	56	0.83 ± 0.6	0.4 ± 0.27	6.41 ± 4.67	0.47 ± 0.28
Ulan Buh Desert	17	0.85 ± 0.61	0.4 ± 0.25	6.41 ± 7.1	0.51 ± 0.26
Alxa Deserts	174	0.76 ± 0.56	0.38 ± 0.25	5.25 ± 4.59	0.49 ± 0.28

Notes: *E* = Pielou evenness index; *S* = Species richness indexes; *H*′ = Shannon–Wiener index; *D* = Simpson index.

**Table 3:** Diversity index of different plant life forms. The data are the average value of different diversity index.

Average Value	Tree	Shrub	Shrub–Herb	Herb
*E*	0.57	0.56	0.48	0.57
*S*	3.64	5.40	5.97	4.50
*H*′	0.55	0.79	0.79	0.71
*D*	0.28	0.39	0.39	0.36

**Table 4:** Characteristics of the 3 desert soil environments. The data are the average value of environment (mean ± SD).

Average Value	Badain Jaran Desert	Tengger Desert	Ulan Buh Desert
SW (%)	2.28 ± 1.49 ^b^	4.61 ± 6.17 ^ab^	6.68 ± 5.88 ^a^
Cly (%)	1.21 ± 0.69 ^b^	2.03 ± 1.72 ^b^	4.81 ± 4.27 ^a^
Sit (%)	5.99 ± 4.96 ^b^	12.03 ± 11.15 ^b^	34.06 ± 33.35 ^a^
Sad (%)	92.79 ± 34.49 ^a^	85.93 ± 42.92 ^a^	61.13 ± 47.77 ^a^
Median size (μm)	186 ± 76 ^a^	141.35 ± 76.91 ^a^	143.55 ± 116.97 ^a^
TN (g/kg)	0.01 ± 0.01 ^a^	0.23 ± 0.16 ^a^	0.16 ± 0.05 ^a^
AP (ppm)	0.33 ± 0.23 ^b^	1.02 ± 0.61 ^a^	1.16 ± 0.56 ^a^
SOM (g/kg)	0.15 ± 0.05 ^b^	0.35 ± 0.26 ^ab^	0.22 ± 0.07 ^a^
EC (μS/cm)	0.07 ± 0.04 ^a^	0.08 ± 0.03 ^a^	0.08 ± 0.01 ^a^
AK (mg/kg)	176.42 ± 68.48 ^b^	141.76 ± 73.88 ^ab^	103.53 ± 20.08 ^a^
RZ (g/cm3)	1.39 ± 0.44 ^a^	1.5 ± 0.1 ^a^	1.55 ± 0.78 ^a^

Notes: Different lowercase letters (^a^,^b^) indicate a significant difference (*p* < 0.05) between different deserts. SW = soil moisture content; Cly = clay content; Sit = powder content; Sad = sand content; Median size = median particle size; TN = total nitrogen; NP = available phosphorus; SOM = soil organic matter; EC = electrical conductivity; AK = available potassium content; AP = available phosphorus; RZ = soil bulk density.

## Data Availability

The data presented in this study are available on request from the corresponding author.

## Supplementary Materials

The following supporting information can be downloaded at: https://www.mdpi.com/article/10.3390/plants12193465/s1, Table S1: Plant Species in Alxa Desert.
