# Supplementary material for: Effect of Soil Environment on Species Diversity of Desert Plant Communities
Source: Plants (Basel). 2023 Oct 2;12(19):3465. doi: 10.3390/plants12193465 (PMC10574983; doi:10.3390/plants12193465)
Supplement: Supplementary file 1 [file plants-12-03465-s001.zip › plants-2614729-supplementary.pdf]

Table S1. Plant Species in Alxa Desert

Serial Number	Families	Genera	Species
1	Plumba ginaceae	Limonium Mill.	Limonium aureum (L.) Hill
2		Limonium Mill.	Limonium aureum
3	Liliaceae	Allium	Allium mongolicum regel
4		Allium	Allium polyrhizum turcz. Ex Regel
5		Allium	Allium condensatum
6		Asparagus	Asparagus gobicus Ivan. ex Grubov
7	Primula ceae	Glaux	Glaux maritima L
8	Tamaric aceae	Reaumuria	Reaumuria soongarica
9		Tamaricaceae	Tamarix chinensis Lour.
10	Labiatae	Panzeria Moench	Panzeria alaschanica kupr
11	Legumi nosae	Astragalus	Astragalus linn
12		Astragalus	Astragalus complanatus
13		Glycyrrhiza	Glycyrrhiza uralensis Fisch
14		Astragalus	Astragalus membranaceus (Fisch.) bunge
15		Oxytropis	Oxytropis glabra DC
16		Caragana	Caragana korshinskii Kom.
17		Astragalus	Astragalus alashan variant
18		Astragalus	Astragalus adsurgens Pall.
19		Astragalus	Astragalus alxanensis
20		Oxytropis	Oxytropis aciphylla Ledeb
21		Ammopiptanthus	Ammopiptanthus mongolicus
22		Sophora	Sophora alopecuroides L.
23		Astragalus	Astragalus sulcatus Linn

24		Astragalus	Astragalus mongholicus Bunge
25		Astragalus	Astragalus variabilis Bunge ex Maxim
26		Hedysarum	Hedysarum scoparium Fisch. & C. A. Mey.
27		Thermopsis	Thermopsis lanceolata
28		Oxytropis	Oxytropis glabra DC
29		Caragana	Caragana stenophylla Pojark
30	Poaceae	Phragmites	Phragmites australis (Cav.) Trin. ex Steud.
31		Psammochloa	Psammochloa villosa (Trin.) Bor
32		Leymus	Leymus secalenus (Georgi) Tzrek
33		Festuca	Festuca ovina L
34		Stipa	Stipa capillata L
35		Achnatherum	Achnatherum splendens
36		Agropyron	Agropyron cristatum (L.) Gaertn
37		**Cleistogenes Keng**	Cleistogenes Keng
38		Eragrostis	Eragrostis pilosa (L.) Beauv.
39		Stipa	Stipa Linn
40		Stipa	Stipa Linn.
41		Cleistogenes	Cls
42		Enneapogon	nneapogon borealis (Griseb.) Honda
43		Stipa	Stipa breviflora Griseb
44		Aristida	Aristida adscensionis L
45		Stipa	Stipa glareosa P.A. Smirn.
46		Blysmus	Brylkinia caudata
47		Puccinellia	Puccinellia tenuiflora (Griseb.) Scribn
48		Pennisetum	Pennisetum centrasiaticum Tzvel
49		Chloris	Chloris virgata Sw

50		Calamagrostis	Calamagrostis epigeios (L.) Roth
51		Phragmites	Phragmites australis (Cav.) Trin. ex Steud.
52		Setaria	Setaria viridis (L.) P. Beauv
53		Stipa	Stipa tianschanica Roshev
54	Elaeagnaceae	Elaeagnus	Elaeagnus angustifolia
55	Zygophyllaceae	Nitraria	Nitraria tangutorum Bobrov
56		Sarcozygium	Sarcozygium xanthoxylon Bunge
57		Tribulus	Tribulus terrestris L.
58		Peganum	Peganum harmala L.
59		Nitraria	Nitraria sphaerocarpa Maxim.
60		Peganum	Peganum nigellastrum Bunge
61		Zygophyllum	Zygophyllum fabago
62		Nitraria	Nitraria tangutorum Bobrov
63		Zygophyllum	Zygophyllum mucronatum
64		Zygophyllum	Zygophyllum potaninii
65	Apocynaceae	Apocynum	Apocynum venetum
66		Cynanchum	Cynanchum cathayense
67		Poacynum	Poacynum hendersonii
68		Poacynum	Poacynum pictum (Schrenk) Baill
69	Compositae	Stilpnolepis	Stilpnolepis centiflora
70		Artemisia	Artemisia arenaria
71		Artemisia	Artemisia sacrorum
72		Sonchus	Sonchus oleraceus L
73		Taraxacum	Taraxacum mongolicum
74		Heteropappus	Heteropappus hispidus
75		Acroptilon	Acroptilon repens (L.) DC.

76		Ajania	Ajania fruticulosa
77		Scorzonera	Scorzonera austriaca Willd
78		Ajania	Ajania pallasiana (Fisch. ex Bess.) Poljak
79		Cirsium	Cirsium Mill. emend. Scop
80		Carduus	Carduus crispus L.
81		Echinops	Echinops gmelinii Turcz.
82		Scorzonera	Scorzonera mongolica Maxim
83		Inula	Inula salsoloides (Turcz.) Ostenf
84		Artemisia	Artemisia vestita Wall
85		Asterothamnus	Asterothamnus centrali-asiaticus Novopokr.
86		Artemisia	Artemisia ordosica Krasch
87		Artemisia	Artemisiadalai–lamaeKrasch
88		Mulgedium	Mulgedium tataricum
89		Saussurea	Saussurea japonica
90		Artemisia	Artemisia salsoloides
91		Taraxacum	Taraxacum sinicum
92		Saussurea	Saussurea runcinata DC
93		Youngia	Youngia tenuicaulis
94		Artemisia	Artemisia xerophytica Krasch.
95		Ixeris	Ixeris denticulata
96		Ajania	Ajania tenuifolia (Jacquem.) Tzvel.
97		Scorzonera	ScorzoneradivaricataTurcz.
98		Neopallasia	Neopallasia pectinata
99		Ixeridium	Ixeridium sonchifolium (Maxim.) Shih
100		Cichorium	Cichorium intybus L

101		Cirsium	Cirsium setosum
102		Heteropappus	Heteropappus altaicus (Willd) Novopokr
103		Ajania	Ajania achilloides(Turcz.)
104		Sonchus	Sonchus arvensis L
105		Artemisia	Artemisia blepharolepis Bunge
106		Artemisia	Artemisia scoparia Waldst. et Kit
107		Artemisia	Artemisia Linn. Sensu stricto, excl. Sect. Seriphidium Bess.
108		Olgaea	Olgaea leucophylla
109	Chenop odiaceae	Corispermum	Corispermum patelliforme Iljin
110		Bassia	Bassia dasyphylla
111		Agriophyllum	Kirilowia eriantha Bunge
112		Salsola	Salsola ruthenica Iljin
113		Agriophyllum	Agriophyllum squarrosum (L.) Moq.
114		Suaeda	Suaeda salsa
115		Haloxylon	Haloxylon ammodendron (C. A. Mey.) Bunge ex Fenzl
116		Salsola/Halogeton	Halogeton arachnoideus Moq
117		Salsola	Salsola arbuscula Pall
118		Salsola	Salsola collina
119		Kalidium	Kalidium foliatum
120		Kalidium	Corispermum mongolicum Iliin
121		Salsola	Salsola passerina Bunge
122		Kalidium	Kalidium cuspidatum (Ung.-Sternb.) Grubov
123		Halogeton	Halogeton glomeratus (M. Bieb.) Ledeb.
124		Ceratoides	Ceratoides latens (J. F. Gmel.) Reveal & N. H. Holmgren
125		Salsola	Salsola laricifolia Turcz. ex Litv
126		Cornulaca	Cornulaca alaschanica C. P. Tsien & G. L. Chu

127		Sympegma	Sympegma regelii Bunge
128		Anabasis	Anabasis brevifolia C．A．Mey
129		Suaeda	Suaeda glauca (Bunge) Bunge
130		Suaeda	Suaeda crassifolia Pall.
131		Suaeda	Suaeda corniculata Bunge
132		Kochia	Kochia scoparia (L.) Schrad
133		Kalidium	Slenderbranch Kalidium
134		Suaeda	Suaeda przewalskii
135		Suaeda	Suaeda glauca Bunge
136		Kalidium	Kalidium cuspidatum (Ung.-Sternb.) Grubov var. sinicum A. J. Li
137		Kalidium	Kalidium foliatum
138	Polygon aceae	Atraphaxis	Atraphaxis bracteata Losinsk
139		Calligonum	Calligonum mongolicum Turcz.
140		Rheum	Rheum palmatum L
141		Calligonum	Calligonum mongolicum
142		Atraphaxis	Atraphaxis frutescens
143	Asclepia daceae	Cynanchum	Cynanchum hancockianum
144		Cynanchum	Cynanchum thesioides (Freyn) K. Schum
145		Cynanchum	Cynanchum chinense R. Br.
146		Periploca	Periploca sepium
147	Ephedra ceae	Ephedra	Ephedra sinica Stapf
148		Ephedra	Ephedra intermedin Schrenk
149		Ephedra	Ephedra przewalskii Stapf
	Verbena ceae	Caryopteris	Caryopteris mongholica Bunge
151		Verbena officinalis	Verbena officinalis L
152	Gerania ceae	Erodium	Erodium stephanianum Willd.

153	Casuarinaceae	Halerpestes	Halerpestes ruthenica (Jacq.) Ovcz
154	Ephedraceae	Halerpestes	Casuarina equisetifolia Forst
155	Cyperaceae	Casuarina	Carex enervis C. A. Mey.
156	Vitaceae	Euphorbia	Parthenocissus tricuspidata
157	Rosaceae	Potaninia	Potaninia mongolica Maxim
158		Amygdalus	Amygdalus mongolica (Maxim.) Ricker
159	Solanaceae	Lycium	Lycium ruthenicum Murray
160		Lycium	Lycium barbarum L
161	Umbelliferae	Ferula	Ferula teterrima H. Karst. & Kir.
162	Cyperaceae	Carex	Carex appendiculata
163		Carex	Carex spp
164		Carex	Carex duriuscula
165	Cruciferae	Pugionium	Pugionium cornutum (L.) Gaertn.
166	Caryopyllaceae	Dianthus	Caryophyllaceae
167		Gymnocarpos	Gymnocarpos przewalskii
168	Juncaginaceae	Triglochin	Triglochin palustre
169	Asparagaceae	Asparagus	Asparagus cochinchinensis（Lour.）
170	Convolvulaceae	Convolvulu	Convolvulus gortschakovii Schrenk
171		Convolvulu	Convolvulus tragacanthoides Turcz
172	Salicaceae	Populus	Populus euphratica
173		Populus	Populus simonii Carr
174		Salix	Salix matsudana Koidz
175		Populus	
176	Rosaceae	Hypecoum	Hypecoum L
177	Ulmaceae	Ulmus	Ulmus pumila L

178	Iridacea e	Iris	Iris lactea Pall. var. chinensis (Fisch.) Koidz
179		Iris	Iris tenuifolia Pall
180	Boragin aceae	Arnebia	Arnebia Forsk.
181		Arnebia	Arnebia fimbriata
182		Tournefortia	Tournefortia sibirica
183	Bignoni aceae	Incarvillea	Incarvillea sinensis Lam
